# An enhanced expression of hypothalamic neuronal nitric oxide synthase in a rat model of simulated transport stress

**DOI:** 10.1186/s12917-019-2071-x

**Published:** 2019-09-06

**Authors:** Jia Wang, Jiao Li, Mingyuan Yu, Yuying Wang, Yunfei Ma

**Affiliations:** College of Veterinary Medicine, China Agricultural University, Beijing, 100193 China

**Keywords:** NO, nNOS, Transport stress, Hypothalamic, Paraventricular nucleus, Supraoptic nucleus

## Abstract

**Background:**

Transport stress not only causes physiological changes but also induces behavioral responses, including anxiety-like and depression-like behavioral responses in animals. The neuronal nitric oxide synthase (nNOS) plays a pivotal role in transport stress. This study aimed to investigate the effects of acute transport stress on the expression of nNOS and the distribution of nNOS-positive neurons in the paraventricular nucleus (PVN) and supraoptic nucleus (SON) of the hypothalamus in rats and to explore the neuroendocrine mechanism of transport stress.

**Results:**

In this study, for the first time, we investigated the effects of transport stress on nitric oxide (NO)-NOS in the hypothalamus. After simulated stress, rats exhibited behavioral changes in the open field test (OFT), increased serum corticosterone (CORT) and norepinephrine (NE) levels, and increased NO content in the hypothalamus. In addition, nNOS expression in the hypothalamic PVN was upregulated, and its distribution was altered in stressed rats compared with that of unstressed rats.

**Conclusions:**

Our findings indicate that simulated transport stress increases nNOS expression and alters its distribution in the PVN of the rat hypothalamus.

## Background

Live animal transport has increased steadily in recent decades. Numerous studies have shown that productive performance, behavioral status, and physiological indicators change after transportation. During transportation, animals are stimulated by a variety of stressors, including capture, vibration, collision, temperature extremes, thirst, and hunger [1]. Superposition of these stressors leads to changes in blood composition, hormones, metabolites, enzymes, and behavior [1, 2]. These changes adversely affect animal health and welfare [3, 4]. In addition, the influence of stressors on animal physiology and behavior may lead to weight loss, increased mortality, and declined meat and skin quality, resulting in significant economic losses [5, 6].

Experimental animals are frequently transported in large quantities to keep up with current developments in scientific research across a wide array of disciplines. The stress on animals caused by transportation from the breeding base to the research institution may induce adverse effects in each transported animal [7]. Behavior, blood biochemical indexes, physiology, and neurohormones of transported animals all change correspondingly [2, 8, 9]. When animals are stressed, the hypothalamic–pituitary–adrenal (HPA) axis is activated and releases corticotropin-releasing hormone (CRH) from the hypothalamus, which promotes the release of adrenocorticotropic hormone (ACTH) in the anterior pituitary. ACTH activates the release of glucocorticoids from the adrenal cortex, which, in turn, produces negative feedback on the hypothalamus, anterior pituitary, and hippocampus to counteract the effects of stress on the internal environment. Notably, neuronal nitric oxide synthase (nNOS) is expressed in immature and mature neurons [10, 11], astrocytes, cerebral arterial outer membranes, and myocardial cells in rats [12, 13]. Nitric oxide (NO), which is primarily produced by nNOS in neural tissues [14, 15], plays a crucial role in the response of these tissues to acute stress. Under stress conditions, NOS regulates sympathetic activation by central CRH and may affect HPA axis activity. The paraventricular nucleus (PVN) is one of the major nuclei in the hypothalamus, which regulates NO activity in stress and is one of the most significant brain regions in terms of nNOS expression [16].

Despite abundant evidence that transport stress affects animal behavior and physiological indicators, the effects of transport stress on NO-NOS in the hypothalamus have not yet been explored. In this study, we used a simulated transport stress model to investigate the effects of transport stress on anxiety-like behavior, NO content, and nNOS expression in the brain, as well as the distribution of nNOS-positive neurons in the PVN and supraoptic nucleus (SON) of the hypothalamus in rats. The aims of this study were to explore the unfavorable effects of transport stress on animals, elucidate the neuroendocrine mechanism of transport stress, and formulate effective therapies to prevent and treat transport stress.

## Methods

### Animals

We used adult male Sprague Dawley rats (200–220 g, Beijing Vital River Laboratory Animal Technology Co. Ltd., Beijing, China) for all experiments in this study. The rats were housed in cages at a moderate density (54 × 39 × 20 cm, *n* = 3) under the following standard laboratory conditions: room temperature (25 °C ± 2 °C), 12-h light/dark cycle, 60% ± 5% humidity, and free access to water and food. Lights were turned on and off at 8:00 a.m. and 8:00 p.m., respectively. The protocols for animal use and experimentation were in accordance with the Beijing Laboratory Animal Welfare and Ethics guidelines. Additionally, all work involving animal research was approved by the Beijing Administration Committee of Laboratory Animals and was in accordance with the China Agricultural University (CAU) Laboratory Animal Welfare and Animal Experimental Ethical Committee guidelines. Every effort was made to minimize the suffering and number of rats used.

### Rat model of simulated transport stress

A rat model of simulated transport stress was established as previously described [17], with a slight modification. The 24 rats used for this model were housed under standard laboratory conditions for 7 days and were randomly divided on the eighth day into a control group (*n* = 12) and a transport stress (TS) group (*n* = 12) based on the random number table method. The rats in the TS group were subjected to vibration at 0.1 g (reactive centrifugal force) on a constant temperature shaker (DHZ-CA, Taicang Co. Ltd., Tianjin, China) from 9:00 a.m. to 11:00 a.m. to simulate transport stress for 2 h.

### Behavioral testing

All rats were subjected to the open field test (OFT) immediately after the simulated transport stress (*n* = 12 rats per group). Rats from the stress and control groups were tested simultaneously so that both groups were tested in the same period. On the day of the test, the rats were transported to the testing room. The testing apparatus was an illuminated, soundproofed box (100 × 100 × 50 cm) with black inner walls. The bottom surface of the box was divided into 25 squares (15 × 15 cm). The apparatus was cleaned with 70% ethanol before testing of each animal. The methodology used for the OFT was the same as that described previously [18]. Each rat was placed onto a corner square of the arena and allowed to explore the open field freely for 5 min per trial. A camera was installed at the top right of the box to record the rats’ activities. The number of squares crossed and the number of rearing episodes (defined as standing on the hindlimbs without touching the wall) were recorded; the sum of these two parameters was used as the total score to evaluate the general activity of the rats.

### Collection and processing of tissue samples

We used a previously described methodology for collection and processing of tissue samples [19]. Six rats in each group were used for molecular biology analysis, and the remaining rats were used for histological analysis. The blood samples were collected 2 h after the final behavioral test. Six rats from each group were anesthetized with 1% sodium pentobarbital (5 mg/100 g body weight) via intraperitoneal injection, exsanguinated via the jugular vein, and then euthanized through neck dislocation. The blood samples were centrifuged at 2000×*g* for 10 min at 4 °C, and then the serum samples were collected and stored at − 20 °C for the determination of corticosterone (CORT) and norepinephrine (NE) contents. After euthanizing the rats, hypothalamic tissues were removed, frozen in liquid nitrogen, and stored at − 80 °C for until subsequent measurements of NO content and nNOS mRNA and protein expression. The other six rats in each group were anesthetized with sodium pentobarbital, perfused transcardially with 200 mL of 5-mM sodium phosphate (pH 7.4)-buffered 0.9% (w/v) saline (PBS), followed by perfusion with 300 mL of 4% (w/v) formaldehyde in 0.1 M of sodium phosphate buffer (pH 7.4). The brains were removed, cut into several blocks, and postfixed with the same fixative for 1 day at 4 °C. After cryoprotection with 30% (w/v) sucrose in PBS overnight, the blocks were embedded with embedding medium on a freezing microtome at − 20 °C for 30 min and were then cut into 30-μm-thick sections, which were placed in PBS for subsequent immunohistochemical staining.

### Corticosterone (CORT), norepinephrine (NE), and nitric oxide (NO) analysis

The serum levels of CORT were measured using a radioimmunoassay with a Vitek Immune Diagnostic Assay System (Bio-RAD iMark, Berkeley, California, USA) according to the manufacturer’s instructions by using a CORT assay kit (HY-10063, Beijing Sino-UK Institute of Biological Technology, Beijing, China). The concentrations of serum NE were detected using an enzyme-linked immunesorbent assay (ELISA) according to the manufacturer’s instructions by using a NE assay kit (H096, Nanjing Jiancheng Bioengineering Institute, Nanjing, China). The samples were weighed and then homogenized on ice to detect the content of NO in the hypothalamus. After the solution was centrifuged at 1000×*g* for 10 min, the supernatant was collected, and the NO content was detected using the nitric-acid-reductase method according to the manufacturer’s instructions using a NO assay kit (A012–1-2, Nanjing Jiancheng Bioengineering Institute, Nanjing, China; *n* = 12 rats per group).

### RNA isolation and reverse transcription-polymerase chain reaction

We used the a previously described methodology for RNA isolation and reverse transcription-polymerase chain reaction [19]. Total RNA was isolated using TRIzol reagent (Invitrogen, Carlsbad, CA, USA) in accordance with the manufacturer’s protocol, followed by purification using an RNeasy mini kit (Qiagen, Valencia, CA, USA). The quality of the purified RNAs was determined by calculating the ratio of the absorbance at 260 and 280 nm using a NanoDrop ND-1000 spectrophotometer (NanoDrop Technologies, Wilmington, DE, USA). RNAs were reverse transcribed into cDNA using a FastQuant RT kit (Tiangen Biotech Co. Ltd., Beijing, China). The expression levels of nNOS were quantified using a semiquantitative reverse transcription-polymerase chain reaction (RT-PCR). Gene expression was normalized with glyceraldehyde-3-phosphate dehydrogenase (GAPDH) as the internal standard. The primer sequences of *nNOS* and *GAPDH* genes are listed as follows: *nNOS-*forward: 5′-AATGGAGACCCCCCTGAGAAC-3′; *nNOS-*reverse: 5′-TCCAGGAGGGTGTCCACCGC-3′; *GAPDH*-forward: 5′-GAAGGTCATCCATGACAACTTTG-3′; *GAPDH*-reverse: 5′-GTCCACCACCCTGTGGTGTAG-3′. The cycling parameters used for amplification were as follows: initial heat denaturation at 95 °C for 5 min; 30 cycles of 94 °C for 45 s, 55 °C for 30 s, and 72 °C for 30 s; and an extension at 72 °C for 7 min (*n* = 6 rats per group).

### Protein extraction and western blotting

Total protein was extracted using a total protein extraction kit (Biochain, Hayward, CA, USA) and was quantified using a bicinchoninic acid protein assay kit (78,510, Pierce, Rockford, IL, USA). A 10% separating gel was prepared and consisted of the following: distilled water, 6.1 mL; 30% acrylamide, 5 mL; 4× sodium dodecyl sulfate-polyacrylamide gel electrophoresis (DS-PAGE) separating gel buffer, 3.75 mL; 10% ammonium persulfate, 0.15 mL; and TEMED, 6 μL. The 10% separating gel was allowed to solidify for 30 min. A 5% stacking gel was prepared (distilled water, 3.2 mL; 30% acrylamide, 0.5 mL; 4× SDS-PAGE separating gel buffer, 1.25 mL; 10% ammonium persulfate, 0.05 mL; and TEMED, 5 μL), and allowed to solidify for 30 min. Next, 20 μg of protein from each sample was loaded on SDS-PAGE sample loading buffer (P0015; Beyotime Institute of Biotechnology, Jiangsu, China). Subsequently, electrophoresis was performed at 80 V for 30 min, and was then switched to 120 V for 90 min. A sponge cushion, filter paper, gel, polyvinylidene fluoride membrane, filter paper, and sponge cushion were placed in the clamp in sequential order and were then placed in 1× transmembrane buffer at 200 mA for 120 min. A polyvinylidene fluoride membrane was washed three times with TBST (10 min each time) and was then placed in a 5% non-fat milk sealing solution, which was placed on a shaking table at room temperature for 2 h. Subsequently, the membranes were probed with a specific rabbit polyclonal antibody against nNOS (1:2500; AB76067, Abcam, Cambridge, MA, USA) overnight at 4 °C. The membranes were then incubated with horseradish peroxidase-conjugated goat antibody against rabbit IgG (1:5000; AP132P, Beijing ComWin Biotech Co., Ltd., Beijing, China) for 1 h at 37 °C. Blots were normalized using β-actin antibody (1:5000; 50,201, Kemei Borui Technology Co., Ltd., Beijing, China) to correct for differences in the loading quantity of the protein samples. One milliliter of electrochemiluminescence liquid (WBKLS0100, Millipore, Billerica, MA, USA) was added onto the protein bands, which were imaged using a Tanon chemiluminescence system (5200, Tanon Science Technology Co., Ltd., Shanghai, China). Densitometric values of immunoblot signals were obtained from three separate experiments (*n* = 6 rats per group) using ImageJ (National Institutes of Health, New York, NY, USA) [19].

### Immunohistochemical staining

For immunohistochemical staining, we used a method that has been described previously [19]. Frozen sections were rinsed in PBS and were then incubated in 2% hydrogen peroxide for 20 min to remove endogenous peroxidase reactivity. All incubations were performed at room temperature, and the sections were rinsed with PBS containing 0.3% Triton X-100 (PBS-X). The sections were incubated overnight with a sheep polyclonal antibody against nNOS (1:1000; ab1529, Millipore, Billerica, MA, USA) in PBS-X containing 0.12% lambda-carrageenan, 0.02% sodium azide, and 1% donkey serum (PBS-XCD), and were then incubated for 2 h with biotinylated donkey anti-sheep IgG (1:100; 711,065,147, Jackson, Penn, Lancaster, USA) in PBS-XCD. Subsequently, the sections were incubated in ABC-peroxidase solution (1:100; PK-6102, Vector, Torrance CA, USA) for 30 min, and the bound peroxidase was finally developed to yield a brown reaction product via reaction for 20–30 min with 0.02% diaminobenzidine-4HCl (DAB; D5637, Sigma Chemical Co, St. Louis, Missouri, USA), and 0.0001% H_2_O_2_ in 50 mM Tris-HCl (pH = 7.6). All stained sections were mounted onto gelatinized glass slides, dried, dehydrated in an ethanol series, cleared in xylene, and coverslipped. The same procedure was carried out for the negative control sections, except that the primary antibodies were substituted with PBS. The sections were observed (Fig. 4) under a microscope (Ni-U, Nikon, Japan), and the immunoreactivity intensities of nNOS were determined using Image-Pro plus 6.0 (Media Cybernetics, Bethesda, MD, USA). The cytoarchitectonic areas of hypothalamic PVN and SON were determined using the rat brain atlas [20, 21], (*n* = 6 rats per group).

### Statistical analysis

Statistically, no less than six rats in each group were required for statistical significance, and the animal experimental design followed the “3R principle” [17]. All data were analyzed using one-way analysis of variance with SPSS 17.0 (IBM Inc., Chicago, IL, USA). Before analysis, the normal distribution of all data was verified using the skewed Kurtosis test. All results are presented as the mean ± SD. Comparisons between the groups were analyzed using a Student’s *t*-test. A *P* < 0.05 was considered statistically significant (*P < 0.05, **P < 0.01).

## Results

### Simulated transport-induced stress responses in rats

**Fig. 1:**
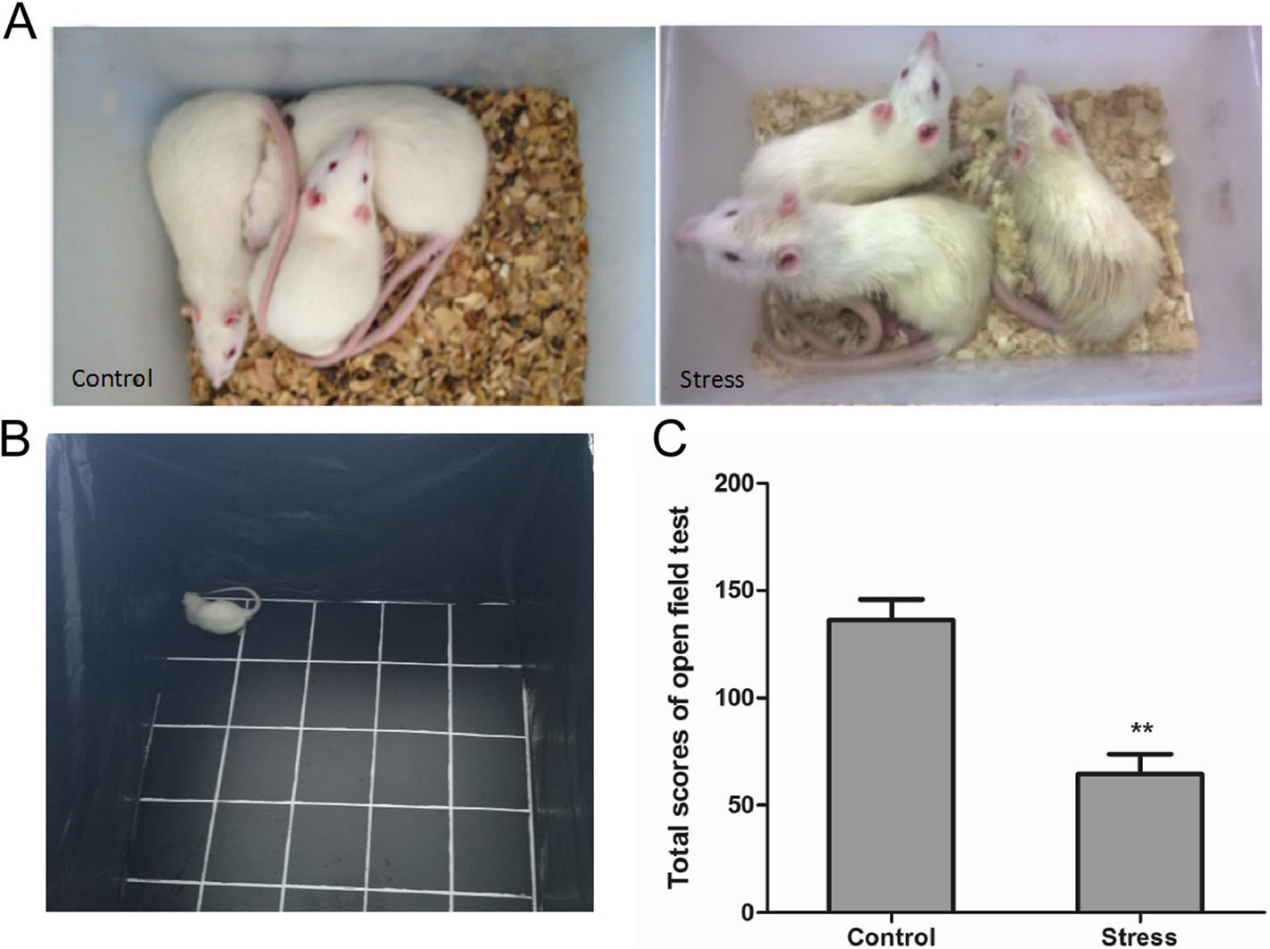
Effect of simulated transport stress on the behavior of rats according to the open-field test. Control and Stress stand for the control group and simulated transport-stress group, respectively. Effect of simulated transport stress on the behavior of rats (**a**) (**b**) and the open-field test scores in each group (**c**). Data are presented as mean ± SD (*n* = 12 rats per group). Comparisons between the groups were analyzed using Student’s *t*-tests. ** stands for the comparison between the control group and simulated transport stress group (*P* < 0.01)

Compared with that of the control group, the rats in the TS group showed abnormal behavior—such as loss of interest, higher sensitivity, matted fur, and rough hair—toward the corner of the open field (Fig. 1a and b). The general activity of the rats was measured using the OFT. The total score of the OFT (Fig. 1c) was significantly lower for the TS group than for the control group (*P* < 0.01).

### Transport stress enhances CORT and NE content in the rat brain

**Fig. 2:**
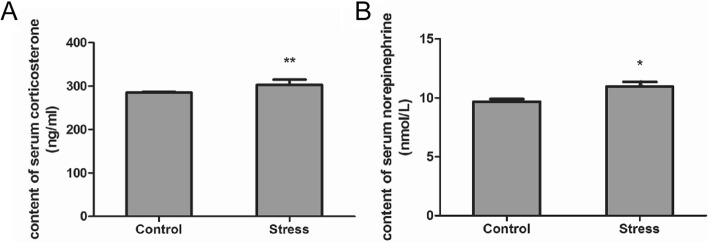
Effect of simulated transport stress on the content of serum CORT and NE in rats. Control and Stress stand for the control group and simulated transport stress group, respectively. Effect of simulated transport stress on the content of serum CORT (**a**) and serum NE (**b**). Data are presented as mean ± SD (*n* = 12 rats per group). Comparisons between the groups were analyzed using Student’s *t*-tests. * stands for the comparison between the control group and simulated transport stress group (*P* < 0.05)

The contents of serum CORT and NE in the rats from each group were analyzed (Fig. 2). The results showed that the contents of serum CORT (Fig. 2a) and NE (Fig. 2b) were significantly increased in the TS group (*P* < 0.05, respectively) compared with those in the control group.

### Transport stress enhances NO content in the rat brain and nNOS mRNA and protein expression in the hypothalamus

**Fig. 3:**
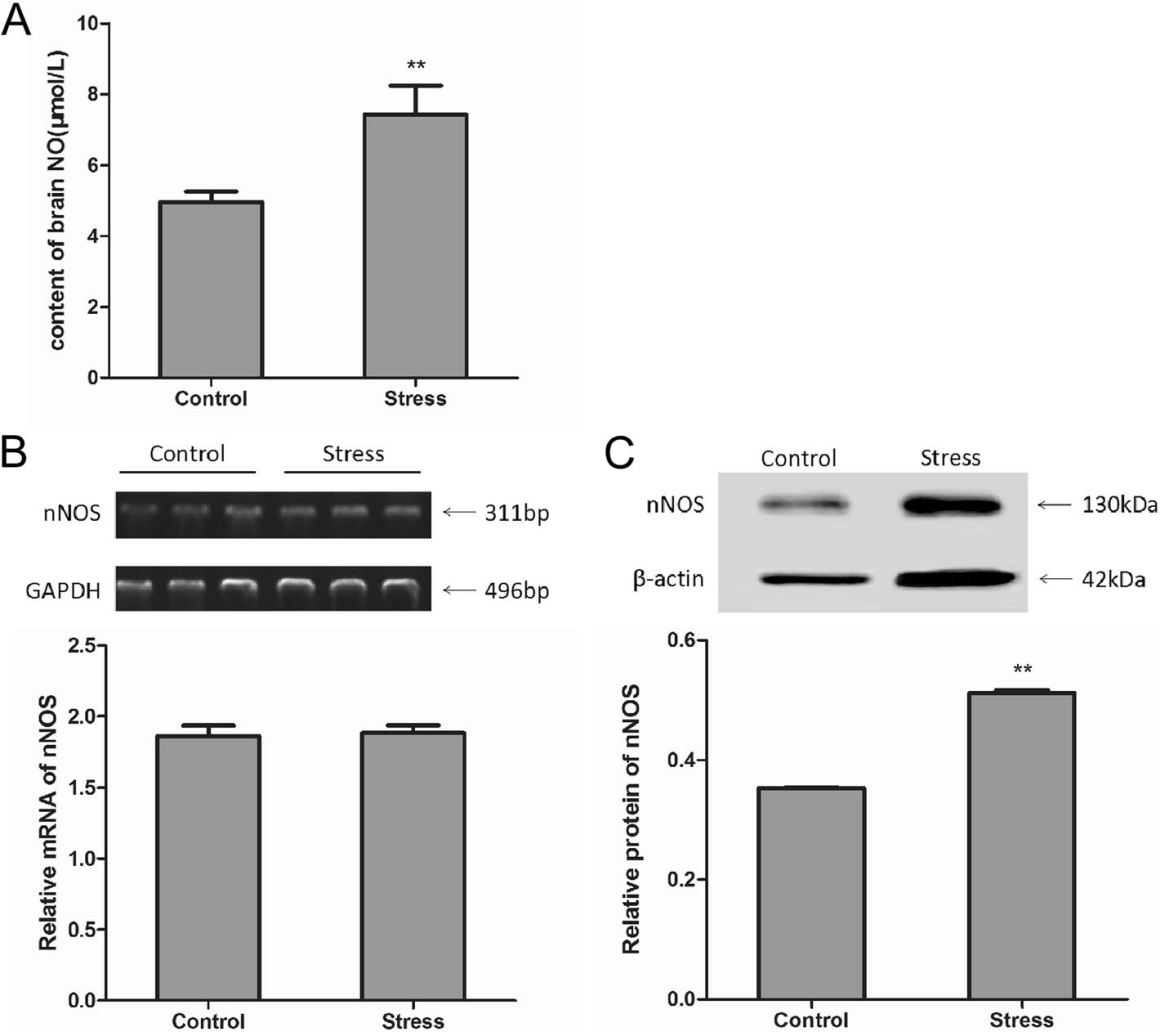
Effect of simulated transport stress on NO and nNOS levels in rats. Control and Stress stand for the control group and simulated transport stress group, respectively. Effect of simulated transport stress on the NO levels (**a**) and nNOS levels (**b**) (**c**). Data are presented as mean ± SD (*n* = 6 rats per group). Comparisons between the groups were analyzed using Student’s *t*-tests. ** stands for the comparison between the control group and simulated transport stress group (*P* < 0.01)

Compared with the control group, the NO content in the brain tissue of the stress group was significantly increased by 49.51% (*P* = 0.006; Fig. 3a). In terms of RT-PCR, no significant difference was observed in the nNOS mRNA expression between the stress and control groups in the hypothalamus (Fig. 3b). Based on the results of western blotting, the expression of nNOS protein in the stress group was significantly increased by 44.92%, as compared with that of the control group (*P* = 0.003; Fig. 3c). These results suggest that stress increases brain NO content and nNOS protein expression.

### Distribution of nNOS-positive neurons in the PVN and SON

**Fig. 4:**
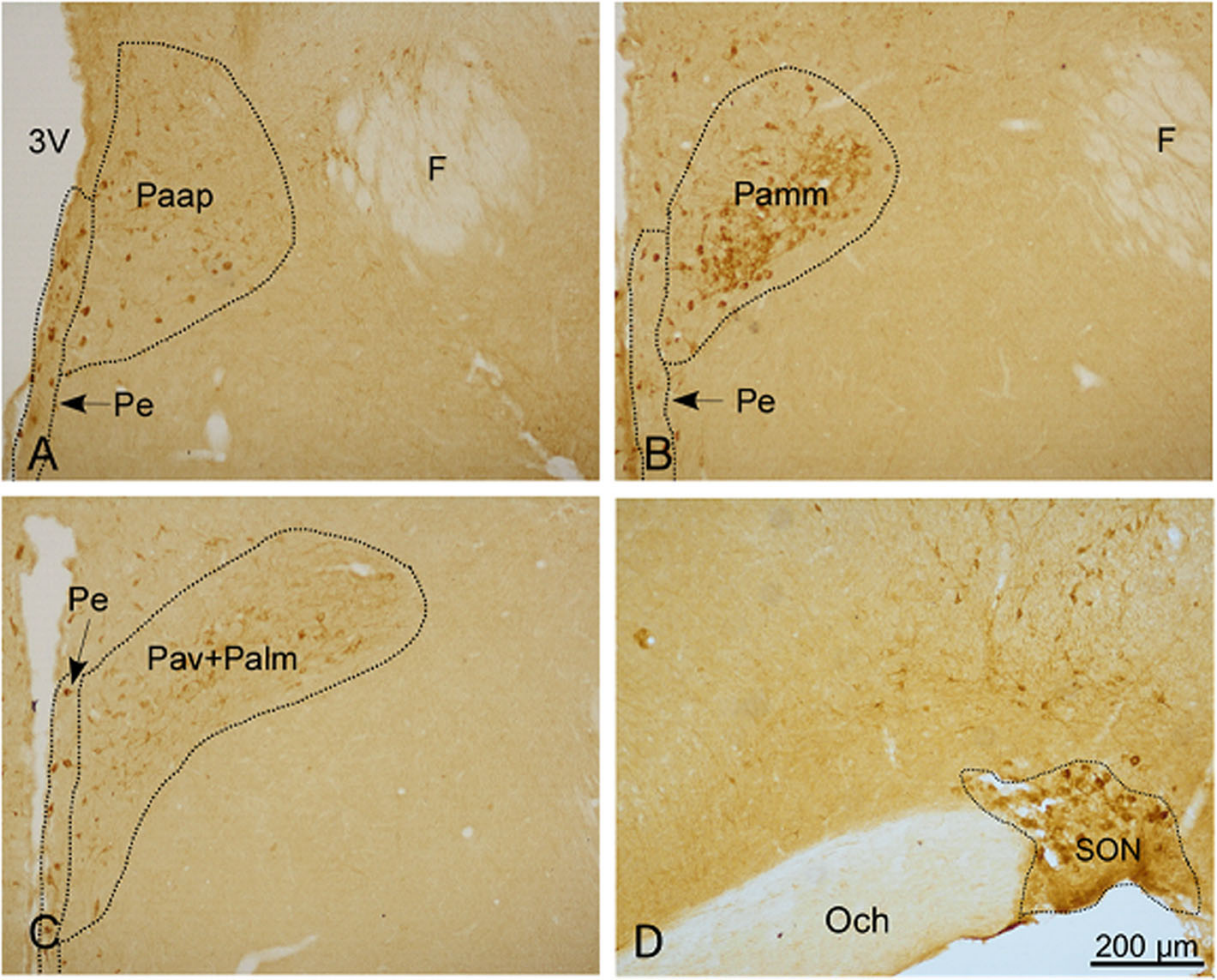
Effect of simulated transport stress on the distribution of nNOS-positive neurons in the PVN and SON. Control and Stress stand for the control group and simulated transport stress group, respectively. Effect of simulated transport stress on the distribution of nNOS-positive neurons in the Paap (**a**), Pamm (**b**), Pav + Palm (**c**), and SON (**d**)

The results of immunohistochemistry showed that (Fig. 4) nNOS-immunopositive neurons were mainly distributed in the PVN and SON of the hypothalamus, primarily in the cytoplasm (with less nuclear staining). The PVN can be divided into the anterior, medial, and posterior subregions. The nNOS neurons were expressed more in the medial and anterior regions, and less in the posterior region. In the anterior chamber of the PVN (Paap), nNOS-positive neurons were scattered, and the cell bodies were fusiform, oval and triangular. The medial part of the PVN is further divided into the medial anterior and medial posterior subregions. The medial anterior part consists mainly of the central large cell area (Pamm) of the hypothalamic PVN. The medial posterior part typically consists of the hypothalamic paraventricular ventral area (Pav) and large cell area (Palm) in the lateral nucleus of the hypothalamus. In the medial part of the hypothalamus, nNOS-positive neurons were densely clustered, and the cell bodies were fusiform, oval and triangular. The cell outlines were clear, and the protrusions were apparent with a deep coloration; the interlacing pattern was indicative of a network structure.

**Fig. 5:**
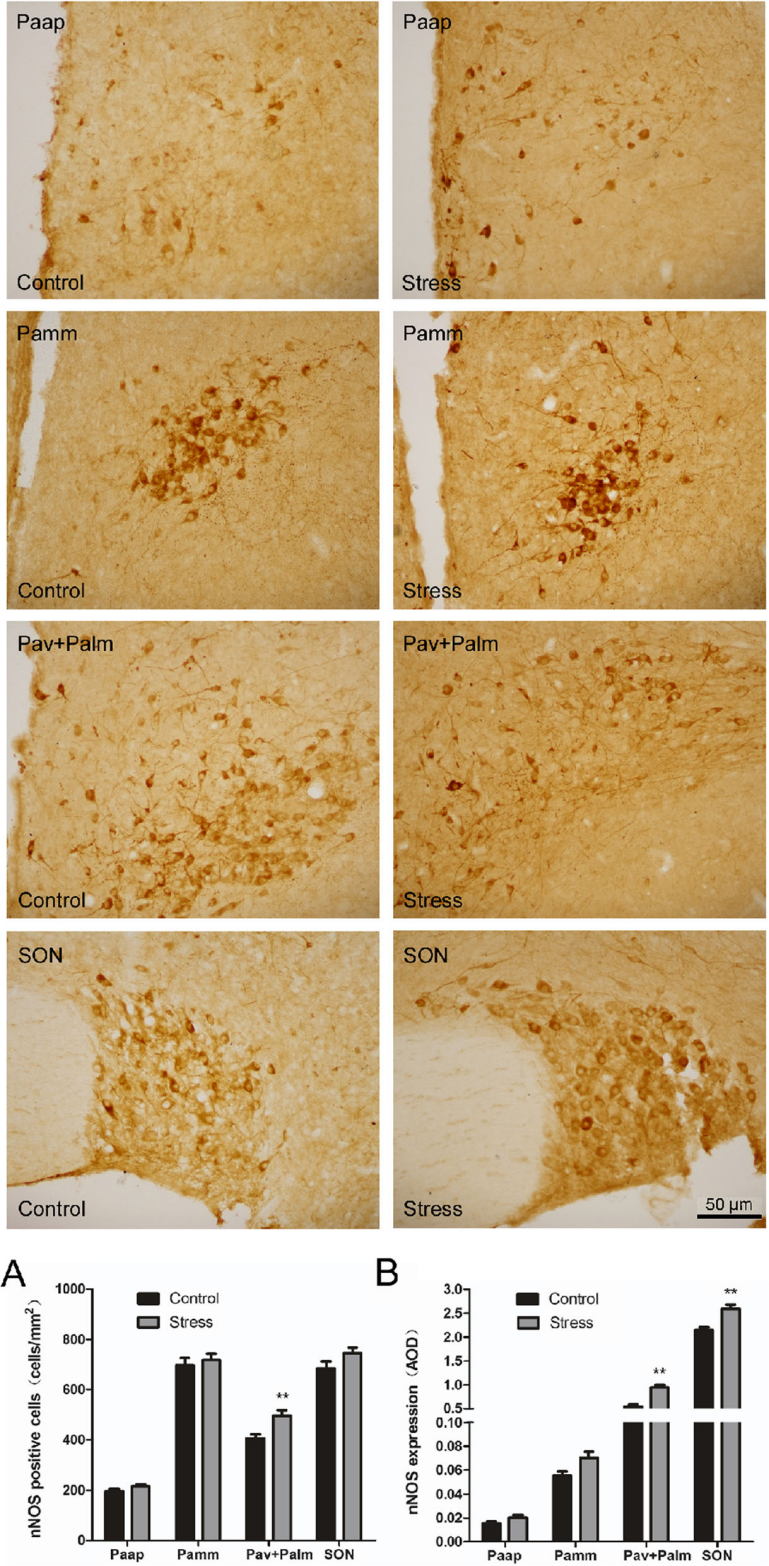
Effect of simulated transport stress on nNOS levels in the hypothalamus of rats. Control and Stress stand for the control group and simulated transport stress group, respectively. Effect of simulated transport stress on nNOS-positive cells (**a**) and nNOS expression (**b**). Data are presented as the mean ± SD (*n* = 6 rats per group). Comparisons between the groups were analyzed using Student’s *t*-tests. ** stands for the comparison between the control group and simulated transport stress group (*P* < 0.01)

We analyzed the number of nNOS-positive cells in the hypothalamus of different treatment groups (Fig. 5). In the Pav + Palm area of the PVN, the number of nNOS-positive cells in the stress group was significantly increased by 22.16% (*P* = 0.001) compared with that of the control group, whereas no significant differences were observed in terms of the numbers of positive cells between the other subregions of the PVN and SON. The average optical density of nNOS-immunoreactive neurons in the hypothalamus of each rat was further analyzed. Compared with that of the control group, the stress group showed increased nNOS expression in the Paap and Pamm areas of the PVN; however, this difference was not significant. nNOS expression in the ventral region of the PVN and the lateral large cell area (Pav + Palm) were higher than those in the first two regions. nNOS expression in the stress group was significantly increased by 73.47% (*P* = 0.001), as compared with that of the control group. nNOS expression in the SON was significantly increased by 20.8% (*P* = 0.001) in the stress group compared with that of the control group.

## Discussion

Animals undergoing long-term transportation will be stimulated by various kinds of stimuli, which will lead to stress, physiological/psychological changes, and even diseases and deaths [22]. Transport stress is a comprehensive stress model, which involves multiple factors [23], such as animal age, gender, transportation distance, and choice of transportation tools. In this study, laboratory animals were subjected on a constant temperature shaker to simulate transport stress. The OFT, as well as serum CORT and NE concentrations, were used to identify whether the model was effective at inducing behavioral and physiological markers of stress. The results showed that the rats in the TS group exhibited lower activity on the OFT and elevated serum CORT. CORT can act as a stress hormone [24, 25]. The OFT is a common method to examine psychological function [26]. In this study, the results of the OFT and the measurement of serum CORT and NE indicated that the transport stress model was successfully established.

The HPA axis is a central control and organ-regulation system that links the central nervous system to the endocrine system. A dysfunction of the HPA axis is related to clinical manifestations of physical and mental illnesses [27]. For example, in severe depression and post-traumatic stress disorder (PTSD), the HPA axis is overactive [28], which has been associated with increased susceptibility to infectious diseases and cardiovascular problems. The HPA axis plays a significant role in homeostatic mechanisms involved in stress responses [29]. CORT is a hormonal end product of the HPA axis [30]. Changes in HPA-axis hormones vary among stimulus types and different varieties of rats, and can be used as an index of the intensity of a stressor [31, 32]. The human stress response is underpinned by core stress hormones, which involve the fast-acting autonomic nervous system (which triggers a noradrenergic response, leading to increased heart and respiration rates) and the slower acting HPA axis (which elicits a glucocorticoid response); this response then downregulates sympathetic arousal [33].

Exposure to stressful stimuli is associated with the activation of NOS and generation of NO [34]. NO plays a crucial role in the regulation of the central nervous system and endocrine function. The presence of NOS in hypothalamic nuclei, median bulges, and the pituitary gland is highly correlated with the regulation of pituitary activity [35]. Previous studies indicate that NO affects the activity of the HPA axis [36], and there is evidence that NO can affect the release of CRH through paracrine actions [37], thereby suggesting that after stress, endogenous NO production activates nNOS, possibly by affecting CRH and activating the HPA axis; this subsequently prompts endogenous production of CORT and catecholamines to resist stress. In this study, the results of western blotting and RT-PCR showed that the expressions of NO and nNOS were increased in the PVN of rats with transport stress, indicating endogenous nNOS activation to produce NO after transport stress.

The hypothalamus occupies the third ventricle on the ventral side and is located above the pituitary. Elevated HPA activity is associated with altered functions of the hypothalamus, pituitary, and gonads [38]. The hypothalamic PVN and SON are closely related to stress [39]. These areas can regulate water/salt balance, blood pressure, and metabolism with the secretion of vasopressin (VAS) and oxytocin (OXY). The activation of the HPA axis relies on the excitation of neuroendocrine neurons in the hypothalamic PVN that secrete CRH [40]. Among them, the hypothalamic PVN, which innervates the median ridge, is the most typical hypothalamic nucleus that secretes CRH and contains neurons that directly innervate the preganglionic autonomic and anterior pituitary. Therefore, the PVN plays a significant role in regulating hypothalamic stress, feeding, drinking behavior, and a series of autonomic responses [41, 42]. The PVN consists of large- and small-cell regions, and each branch has a sub-branch that contains a unique neurotransmitter distribution [43]. The hypothalamic PVN is a complete and unique part of the neuroendocrine system. Concerning structural function, the PVN can be divided into three main types of neurons: large neuroendocrine neurons, small neuroendocrine neurons, and descending neurons [44, 45]. Large neuroendocrine neurons are classified into VAS and OXY neurons. Small neuroendocrine neurons are divided into the neurons that synthesize and release CRH, thyrotropin-releasing hormone (TRH), dopamine, somatostatin (SS), and gonadotropin-releasing hormone. Almost all of the neuroendocrine neurons that synthesize and release CRH and TRH in the brain are in the PVN, as are most SS endocrine neurons. Descending PVN neurons directly innervate the thalamus, brainstem, and spinal cord. In this study, the PVN was divided into four regions for analysis: the Paap, Pamm, Pav, and Palm. The results showed that nNOS-positive neurons in the control group were distributed in different regions of the PVN and were highly distributed in the ventral region and the lateral large cell region (Pav + Palm). Taken together, this study explored the effects of transport stress on nNOS expression in the hypothalamic PVN and the distribution of this morphology in rats. The findings from this study provide a basis for the investigation of the relationship among the hypothalamus, NO-nNOS, and the HPA axis.

## Conclusions

The current study used a rat model of simulated transport stress to investigate the effects of transport stress on nNOS expression in the rat brain, especially in regions related to stress responses, such as the hypothalamus. Physiological and psychological stress responses were successfully induced in rats subjected to simulated transport stress. Transport stress enhanced nNOS expression in the hypothalamus, indicating that nitrergic neurons in the hypothalamus are likely involved in transportation-induced stress, anxiety, and behavioral dysfunction.

## Data Availability

The data sets analyzed and used in the current study are available from the corresponding author upon reasonable request.

## Abbreviations

CORT: Serum corticosterone
CRH: Corticotropin-releasing hormone
HPA: Hypothalamic–pituitary–adrenal
NE: Norepinephrine
nNOS: neuronal nitric oxide synthase
NO: Nitric oxide
PVN: Paraventricular nucleus
SON: Supraoptic nucleus
